# Evaluation of therapeutic response and tolerability to intravenous iron sucrose and ferric carboxymaltose among pregnant women with iron-deficiency anemia: A 6-year experience in a tertiary care center

**DOI:** 10.3892/mi.2025.233

**Published:** 2025-04-03

**Authors:** Govind R. Patel, Indu Thanvi, Ramesh Chandra Seervi, Ramesh Jakhar

**Affiliations:** 1 Department of Clinical Hematology, Dr. SN Medical College, Jodhpur, Rajasthan 342003, India; 2 Department of General Medicine, Dr. SN Medical College, Jodhpur, Rajasthan 342003, India

**Keywords:** iron-deficiency anemia, intravenous iron, iron sucrose, ferric carboxymaltose, pregnancy

## Abstract

Iron-deficiency anemia (IDA) is a global health concern in pregnancy associated with adverse fetal and maternal outcomes. The present study aimed to evaluate and compare the therapeutic response and tolerability of intravenous (IV) iron sucrose and ferric carboxymaltose (FCM) administered to pregnant women with IDA. The present prospective observational study was conducted among 334 pregnant women who were in the second or third trimester of pregnancy with moderate to severe IDA and who were treated with IV iron sucrose or FCM at a large tertiary care center between April, 2018 and March, 2024. The therapeutic response was assessed by analyzing the increase in hemoglobin (Hb) and serum ferritin levels at 3 and 6 weeks following the first dose of IV iron treatment. Tolerability was assessed by analyzing the adverse events to drug administration. A statistically significant increase in the mean Hb and serum ferritin levels was observed in both the iron sucrose and FCM groups at 3 and 6 weeks post-infusion (P<0.0001 for all); however, the increase in the FCM group was significantly higher (P<0.0001) than that in the iron sucrose group. Minor temporary adverse drug reactions were comparable (P=0.232) between the both treatment groups, with no major serious adverse events observed in any group. IV iron sucrose and FCM both were efficacious and well tolerated in pregnant women with moderate to severe IDA during the second and third trimester. However, there was an improved overall response to FCM as it caused a greater increase in the Hb and serum ferritin levels than iron sucrose. Therefore, FCM is recommended as an effective and safe alternative to iron sucrose for the treatment of IDA during pregnancy.

## Introduction

Iron-deficiency anemia (IDA) is the most common cause of anemia encountered during pregnancy worldwide, affecting almost 30% of pregnant women in high-income countries, and increasing to >50% of pregnant women in low-income countries (1). In India, the majority of the women become pregnant with low hemoglobin (Hb) level owing to lack of nutritional iron intake and short pregnancy intervals, resulting in a higher incidence of moderate to severe anemia during pregnancy. In the National Family Health Survey-5, almost 53.7% of Indian pregnant women were found to be anemic (2). IDA during pregnancy is associated with several adverse maternal and fetal outcomes, such as the need for blood transfusion, postpartum hemorrhage (3,4) and cardiovascular issues in the mother (5), an increased risk of babies with a low birth weight, newborns who are small for their gestational age and preterm birth (6), diminished auditory recognition memory in infants (7), and long-term neurocognitive effects in childhood that may persist into adulthood (8,9). This in turn results in higher perinatal, maternal and infant mortality rates (10,11). The trapid correction of anemia and repletion of iron stores is, therefore, required to avoid these complications.

Oral iron therapy is currently considered as the first-line treatment for the majority of patients with IDA (12). However, it can be ineffective due to poor absorption or poor compliance owing to intolerable gastrointestinal side-effects, or it may not facilitate urgent rapid iron repletion, particularly in women with moderate to severe IDA presented too late during pregnancy (13,14). Parenteral iron helps in restoring iron stores at a more rapid rate and more effectively than oral iron. Thus, pregnant women with moderate to severe IDA, particularly in the late second and third trimester should be more effectively treated with parenteral iron therapy. Intramuscular iron injections are painful and have to be repeatedly administered. Intravenous (IV) iron is a safe and effective alternative treatment option for IDA in pregnancy and has been recommended in various guidelines (12,15,16). The IV iron preparations available in India are low-molecular weight iron dextran, iron sucrose, ferric carboxymaltose (FCM) and iron isomaltoside. A test dose is necessary prior to administering IV iron dextran, as severe anaphylactic reactions have been reported with its use. Iron sucrose, FCM and iron isomaltoside have been shown to be efficacious and safe in pregnancy (16,17). A test dose is not required before administrating these newer IV iron formulations. Although both iron sucrose and FCM can be administered without a test dose, multiple doses and prolonged infusion times are typically required with IV iron sucrose (18). FCM has a neutral Ph (5.0-7.0) and physiological osmolarity, which makes it possible to administer its higher single doses over shorter time periods (single dose up to 1,000 mg over a period of 15 min) than other parenteral preparations (19).

Data from observational studies on the therapeutic effects and adverse reactions of these newer IV iron preparations are critical to guide clinical management decisions, and to assure the safety of expecting mother and the unborn fetus. Studies have reported the safe and effective use of iron sucrose (20) and FCM (21,22) in the treatment of IDA during pregnancy. The comparison of the efficacy and safety profile of iron sucrose vs. FCM for the treatment of IDA during pregnancy has been demonstrated in previous research (23). However, there are limited data available regarding the comparative efficacy and safety of IV iron sucrose and FCM administration during pregnancy in India. Therefore, the present study aimed to evaluate and compare the therapeutic response and tolerability of IV iron sucrose and FCM in the treatment of moderate to severe IDA during pregnancy.

## Subjects and methods

### Study design and study population

The present prospective observational study was carried out at the Department of Clinical Hematology, Mathura Das Mathur Hospital, Dr. SN Medical College, Jodhpur, Rajasthan, India among 334 referred pregnant women in the second or third trimester (gestational age, ≥13 weeks) diagnosed with moderate to severe IDA (Hb <10 g/dl) who were treated with either IV iron sucrose or FCM between April, 2018 and March, 2024. All women were administered IV iron therapy due to the lack of a response following oral iron treatment, intolerance to oral iron therapy, or the need for a rapid increase in Hb levels, as these women were too close to term to opt for oral iron treatment. The selection between iron sucrose and FCM was based on affordability and/or the preference of each women. IDA was diagnosed in the case that the Hb values were <11.0 g/dl and the serum ferritin levels were <30 µg/dl. Moderate anemia was defined as Hb 7.0-9.9 g/dl and severe anemia as Hb <7.0 g/dl. Pregnant women with causes of anemia other than IDA, such as sickle cell anemia, thalassemia, aplastic anemia, megaloblastic anemia, anemia due to chronic disease (renal or hepatic disease), a history of recent blood transfusions and a known history of allergy/anaphylaxis to parenteral iron therapy were excluded from the study. Patients who could not be followed-up till the end of the 6-week period following IV iron therapy were also not included in the final analysis.

### Study procedure

The approval of the Institutional Ethics Committee, Dr. SN Medical College, Jodhpur, India (SNMC/IEC/IIP/2018/139) was obtained before commencing the study. A written informed consent was obtained from all participants prior to their enrolment in the study. The patient records and all data used in the present study were fully anonymized prior to being transferred to the SPSS program for data analysis and reporting.

Baseline data on demographic and clinical characteristics were collected. Laboratory data on Hb and serum ferritin values at baseline prior to iron infusion and then again at ~3 and 6 weeks following the first drug infusion were obtained. Blood samples were collected from the antecubital vein into both anticoagulant and plain tubes, and serum was then separated from the clotted blood in the plain tubes through centrifugation at 2,000 x g for 10 min at room temperature (20-25˚C). Hemoglobin was measured using the cyanide-free colorimetric method through a fully automated 5-part differential hematology analyzer (ELite 580, Erba Lachema s.r.o.) and serum ferritin measurement was performed using chemiluminescence immunoassay (CLIA) through biochemistry analyzer (Vitros 3600, Ortho Clinical Diagnostics). Any adverse events during and after IV iron administration were also recorded. Available data from all women were analyzed for the therapeutic response and tolerability of IV iron therapy in pregnancy. The primary outcome measure of the present study was to evaluate the therapeutic response of IV iron sucrose and FCM, which was assessed by analyzing the increase in Hb and serum ferritin levels at 3 and 6 weeks following the first infusion of IV iron. The secondary outcome measure was the maternal tolerability of IV iron therapy, which was assessed by analyzing the adverse effects to drug administration.

### Dose calculation and administration of IV iron

The cumulative doses for IV iron sucrose and FCM required for Hb restoration and the repletion of iron stores were calculated using the formula by Ganzoni (24) and as previously described (25), as follows: Iron requirement (mg)=cumulative iron deficit (mg)=[2.4 x (target Hb - actual Hb) (g/dl) x pre-pregnancy body weight (kg)] + iron storage depot (mg), where, 2.4 is a correction factor that is derived from the blood volume of the patient, estimated at 7% of body weight and the iron content of Hb, which is 0.34% (0.07x0.0034x100=2.4) (conversion from g/dl to mg). The target Hb was taken as 11.0 g/dl during pregnancy as per the WHO criteria (26). Instead of adding iron stores as 15 mg/kg body weight for women <35 kg and 500 mg for women >35 kg body weight, 1,000 mg were added for the replenishment of iron stores owing to very low iron stores among Indian women (15,20). The calculated cumulative dose was to be rounded off to the nearest 100 mg for each individual.

Iron sucrose (Injection Orofer S, Emcure Pharmaceuticals Limited) was administered as an IV infusion in a dose of 200 mg in 200 ml of normal saline (NS) over a period of 30 min on alternate days until the dosage was completed, which was not to exceed 600 mg per week.

FCM (Injection Orofer FCM, Emcure Pharmaceuticals Limited) was administered in a maximum single dose of 1,000 mg diluted in 200 ml NS as an IV infusion over a period of 30 min. A longer infusion protocol (30 min) than recommended by the manufacturer (15 min) was used owing to the limited availability of safety data for its use in pregnancy. The remainder of the doses, if needed, were administered on the 8th and 15th day.

IV iron therapy was administered by nurses under the supervision of a doctor. Women were observed during and 60 min following the infusion for adverse effects, before being discharged home. Any minor or major adverse drug reactions during and after IV iron administration were documented.

### Statistical analysis

All statistical analyses were performed using the Statistical Package for the Social Sciences (SPSS) software version 24.0 for Windows (version 24.0 for Windows; IBM Corp.). For the analysis, the study participants were categorized into two groups based on the type of IV iron they received: Iron sucrose or FCM. The distribution of the data was analyzed using the Kolmogorov-Smirnov test and was found to be normally distributed. Continuous variables are expressed as the mean ± standard deviation (SD), and categorical variables are presented as frequency and percentage. Comparisons between the two groups were performed using the unpaired t-test for continuous variables and Pearson's Chi-squared (χ^2^) test or Fisher's exact test for categorical values, as appropriate. To compare the Hb and serum ferritin levels obtained before and after treatment (at 3 and 6 weeks) within each of the two groups, a paired-samples t-test was applied. A two-tailed P-value <0.05 was considered to indicate a statistically significant difference for all analyses.

## Results

### Demographic and baseline clinical characteristics of the study participants

A total of 390 pregnant women with IDA treated with IV iron during the study period were included in the present study: A total of 208 women received iron sucrose and 182 women received FCM. Of these women, 56 women were lost to follow-up: A total of 32 women from the iron sucrose group and 24 women from the FCM group. Out of the 334 women finally analyzed, 176 (52.7%) belonged to the iron sucrose group and 158 (47.3%) to the FCM group. Overall, 104 (31.1%) women had moderate anemia, while 230 (68.9%) had severe anemia at baseline. The demographic and baseline clinical characteristics of the study population are presented in Table I. Age, body mass index, gestational age, parity, the use of pre-infusion oral iron supplements and the presence of the obstetrical complications were comparable between the two groups (P>0.05). No statistically significant differences were observed in the mean Hb and ferritin levels at baseline (P=0.172 and 0.182, respectively). In addition, there was no significant difference between the two treatment groups at baseline with respect to the grade of anemia (P=0.687). However, a significantly lower total number of doses was required for FCM therapy compared to iron sucrose therapy (P<0.0001).

### Therapeutic response

The changes observed in Hb and ferritin levels over the post-infusion period are depicted in Table II. There was a statistically significant increase from baseline in the mean Hb and serum ferritin levels at 3 and 6 weeks post-infusion in both the iron sucrose and FCM groups (P<0.0001 for all). However, both the mean Hb and ferritin levels exhibited a significantly higher increase in the FCM group compared to the iron sucrose group at both post-infusion time points (P<0.0001 for all).

Despite having the lower mean baseline Hb, 64 (40.5%) women in the FCM group achieved target Hb levels (≥11.0 g/dl) at 6 weeks compared to 48 (27.3%) in the iron sucrose group (P=0.014) (Table II). Hence, FCM proved more effective than iron sucrose at achieving target Hb levels by the end of 6 weeks.

In subgroup analysis for moderate and severe anemia, FCM was found to cause a statistically significant increase in the Hb level compared with iron sucrose in the both grades of anemia at 3 weeks and 6 weeks (P<0.0001 for all) as shown in Table III.

### Tolerability

Adverse events to IV iron therapy are presented in Table IV. Although the frequency of overall adverse effects observed was lower in the FCM group (7%) as compared to the iron sucrose group (11.4%), no statistically significant difference between the both treatment groups was found (P=0.232). The most common adverse event observed in both groups was local pain/burning/irritation at the site of infusion, which was occurred in 13 women (7.4%) in the iron sucrose group compared to 7 (4.4%) women in the FCM group (P=0.365). There were no severe adverse drug reactions or episodes of anaphylactic shock in any of the patients in both groups.

## Discussion

The present study evaluated and compared the therapeutic response and tolerability of IV iron sucrose and FCM in the treatment of moderate to severe IDA among Indian pregnant women who reported to a large tertiary care center at the second or third trimester of pregnancy. The present study strengthened the evidence on the higher therapeutic response and comparable tolerability of FCM as compared to iron sucrose in pregnancy.

Over the past few years, multiple studies have been published on the use of IV iron sucrose and FCM for the treatment of IDA in pregnancy. Kriplani *et al* (20) reported a significant increase in the mean Hb level by 2.27 g/dl after 4 weeks and 3.57 g/dl after 8 weeks in pregnant women receiving IV iron sucrose therapy (P<0.001). In the present study, an increase of 2.26 g/dl and 2.96 g/dl in Hb levels was achieved at 3 weeks and 6 weeks, respectively (P<0.0001 for both) with iron sucrose therapy, irrespective of the grade of anemia. Thus, the results of the present study are equivalent to the aforementioned previous study. Similarly, there was a significant increase in the mean serum ferritin level by 14.4 µg/l after 4 weeks and 57.8 µg/l after 8 weeks of iron sucrose therapy (P<0.001) in the study by Kriplani *et al* (20). In the present study, the mean ferritin level increased by 50.63 µg/l at 3 weeks and 64.58 µg/l at 6 weeks in the iron sucrose group (P<0.0001 for both). Thus, the results of the present study were similar to those in the study by Kriplani *et al* (20). Various studies have also demonstrated a significant increase in the Hb level and the replenishment of iron stores over a period of 3 to 12 weeks in pregnant women with IDA receiving FCM infusion (21,22,27). In the present study, an increase of 2.95 g/dl and 4.01 g/dl in the Hb levels was observed after 3 and 6 weeks, respectively (P<0.0001 for both) with FCM therapy, irrespective of the grade of anemia. In addition, an increase in the mean ferritin level of 79.94 µg/l at 3 weeks and 97.20 µg/l was observed at 6 weeks in the FCM group (P<0.0001 for both). Thus, the results of the present study were consistent with the findings of the aforementioned previous studies.

The study published by Christoph *et al* (23) was the first comparative study on the use of iron sucrose vs. FCM for the treatment of IDA in pregnant women. The authors of that study documented a comparable increase in Hb levels at the end of the study in both the FCM and iron sucrose groups. In another comparative observational study, Jose *et al* (28), by contrast, revealed that treatment with FCM achieved a significantly higher increase in the Hb level and the more rapid replenishment of iron stores compared to iron sucrose over a duration of 12 weeks. Similarly, the studies conducted by Agrawal and Masand (29), Mahaur *et al* (30), Patel *et al* (31) and Parikh and Agarwal (32) demonstrated a significantly higher increase in Hb and serum ferritin levels in pregnant women who received FCM than in pregnant women who received iron sucrose. In the present study, a significant increase in the Hb level by 4.01 g/dl was noted after 6 weeks of treatment with FCM (P<0.0001), which was 1.35-fold greater than the increase noted with iron sucrose (2.96 g/dl). Similarly, a significant increase of 97.20 µg/l in the serum ferritin level was noted after 6 weeks in the FCM group (P<0.0001), which was 1.5-fold greater than the incrase noted in the iron sucrose group (64.58 µg/l), highlighting that FCM replenished iron stores more effectively compared to iron sucrose. Hence, the results from the present study are consistent with the majority of the aforementioned studies, strengthening the evidence on the higher therapeutic response of FCM as compared to iron sucrose. Keeping in mind the very low iron stores in Indian women, 1,000 mg was added for the replenishment of iron stores. Even with this, the maximum mean serum ferritin levels after 6 weeks of therapy with iron sucrose and FCM were within the normal range. The reason for this may be due to the severely depleted iron stores in Indian women.

The main reason for the differences in the response between iron sucrose and FCM is the distinct chemical structure of the iron complexes. FCM has a more stable iron-carbohydrate complex than iron sucrose, which allows for a slower and more controlled release of iron into the bloodstream compared to the more rapid release observed with iron sucrose; this leads to a more sustained and greater increase in hemoglobin levels with larger single doses and fewer required infusions compared to iron sucrose, often translating to better patient compliance and a more efficient treatment for IDA (33).

Adverse reactions can occur with various IV iron preparations. Minor local reactions are the most common adverse effects observed with IV iron therapy. Kriplani *et al* (20) reported adverse effects with IV iron sucrose therapy in 11% of pregnant women. The reported incidence of adverse effects with FCM therapy was 11% by Froessler *et al* (22), 4% by Gupte *et al* (27) and 8.6% by Trivedi *et al* (34). The various comparative studies reported the incidence of adverse events from 3 to 10.7% for iron sucrose and from 0 to 7.8% for FCM among pregnant women (23,29,31). Although FCM was observed to have lesser adverse events in the majority of these studies as compared to iron sucrose, no statistically significant difference between the both groups was noted and they failed to document the safety of any one iron preparation over the other. The majority of the aforementioned previous studies did not report any major adverse event among pregnant women receiving iron sucrose or FCM therapy. Consistent with the findings of these studies (23,29,31), in the present study, minor and transient adverse reactions were experienced by 11.4% of women receiving iron sucrose and 7% of women receiving FCM, with no statistically significant difference between the both treatment groups (P=0.232). All women had an uneventful recovery. Similar to the aforementioned studies (23,29,31), no major adverse effects or anaphylactic reactions were recorded in either group in the present study. Hence, both iron sucrose and FCM were well tolerated in the present study.

Some studies have suggested that FCM may be associated with fewer side-effects, such as injection site reactions compared to iron sucrose (30-32). As a type I complex, FCM delivers iron gradually and mainly to the RES of the liver. This targeted and slow release accounts for the low toxicity of FCM and the large safety margin between normal and lethal dosing. Due to these factors and owing to the neutral pH and physiological osmolarity, relatively high doses of FCM can be administered with good local tolerance (32).

In addition to a significantly higher increase in Hb and serum ferritin levels along with comparable adverse effects with FCM, the present study demonstrated that a lesser total number of doses (convenient dosing) and, hence, less frequent hospital visits were required for FCM therapy compared to iron sucrose therapy attributed to a large dose administration of FCM in a single sitting. This is very useful in pregnant women presenting with moderate to severe anemia, particularly in late pregnancy, as it reduces the discomfort caused to the women due to transport and multiple needle pricks, improving their compliance.

A limitation of the present study was the lack of randomization. The specific modality of treatment (iron sucrose or FCM) for a woman was decided according to her affordability and/or preference. This may have introduced a selection bias in the present study. However, data on adverse effects were reliable, as they were prospectively collected and documented during and after treatment. Another limitation of the present study was that the data regarding neonatal outcomes could not be analyzed owing to the unavailability of data, as all women were referred to the Department of Clinical Hematology, Mathura Das Mathur Hospital, Dr. SN Medical College, Jodhpur, India only for the management of IDA and they then delivered elsewhere. Finally, as a longer follow-up period was not possible, the effects of IV iron treatment on maternal quality of life were not assessed and compared between the two treatment groups. Thus, further large prospective studies and randomized controlled trials are required to compare the therapeutic response, maternal tolerability, maternal quality of life and neonatal outcomes between IV iron sucrose and FCM therapy in the Indian setting. The strength of the present study, however, is that the study clearly demonstrated the advantage of FCM therapy over iron sucrose in more effectively improving anemia and replenishing iron stores in women with IDA during late pregnancy. To the best of our knowledge, this was the first study to significantly assess and compare the therapeutic response and tolerability of IV iron sucrose and FCM treatment for IDA during pregnancy among pregnant women in Western India.

In conclusion, the results of the present study demonstrated that IV iron sucrose and FCM both were efficacious and well tolerated to treat moderate to severe IDA in pregnancy during second and third trimester, with comparable minor adverse reactions and no severe adverse effects. However, FCM proved to be more effective than iron sucrose in the correction of IDA, as well as in the replenishment of iron stores, as the increase in Hb and serum ferritin levels was significantly higher with FCM as compared to iron sucrose. Additionally, less frequent hospital visits were required for FCM therapy compared to iron sucrose therapy owing to a large dose administration per sitting and lesser total number of doses required (convenient dosing). Therefore, the use of IV FCM is recommended as an effective and safe alternative to iron sucrose for the treatment of moderate to severe IDA during second and third trimester of pregnancy.

## Figures and Tables

**Table I:** Demographic and baseline clinical characteristics of the study population.

Characteristic	Iron sucrose (n=176)	FCM (n=158)	P-value^a^
Age (years)	26.1±3.46	25.8±3.67	0.443
BMI (kg/m^2^)	25.2±1.81	25.5±1.96	0.147
Gestational age at first dose (weeks)	25.8±3.8	26.4±3.5	0.136
Parity			
Primipara	79 (44.9)	60(38)	0.243
Multipara	97 (55.1)	98(62)	
Pre-infusion oral iron supplements	83 (47.2)	71 (44.9)	0.767
Associated obstetrical complications			
PIH	23 (13.1)	30(19)	0.184
GDM	16 (9.1)	19(12)	0.487
Multiple pregnancy	14(8)	8 (5.1)	0.399
Placenta previa	19 (10.8)	14 (8.9)	0.683
Previous ≥1 LSCS	20 (11.4)	22 (13.9)	0.589
Baseline Hb (g/dl)	7.05±1.63	6.81±1.57	0.172
Severity of anemia			
Moderate (Hb 7.0-9.9 g/dl)	57 (32.4)	47 (29.7)	0.687
Severe (Hb <7.0 g/dl)	119 (67.6)	111 (70.3)	
Baseline ferritin (µg/l)	14.52±5.22	13.79±4.69	0.182
Number of doses administered			
1-3	44(25)	158(100)	<0.0001
4-6	127 (72.2)	0 (0)	
>6	5 (2.8)	0 (0)	

Data are expressed as the mean ± standard deviation or as number (%).

^a^The unpaired t-test and Chi-squared test were used to assess the significance of differences in continuous and categorical variables, respectively. FCM, ferric carboxymaltose; BMI, body mass index; PIH, pregnancy-induced hypertension; GDM, gestational diabetes mellitus; LSCS, lower segment cesarean section; Hb, hemoglobin.

**Table II:** Changes in hemoglobin and serum ferritin levels over the post-infusion period.

Variable	Iron sucrose (n=176)	FCM (n=158)	P-value^a^
Hemoglobin (g/dl)			
At baseline	7.05±1.63	6.81±1.57	0.172
At 3 weeks	9.31±1.23	9.76±1.86	0.0089
Change from baseline to 3 weeks	2.26±0.48	2.95±0.63	<0.0001
P-value^b^	<0.0001	<0.0001	
At 6 weeks	10.01±0.80	10.82±1.70	<0.0001
Change from baseline to 6 weeks	2.96±0.27	4.01±0.87	<0.0001
P-value^b^	<0.0001	<0.0001	
Hb level ≥11.0 g/dl	64 (40.5)	48 (27.3)	0.014
Serum ferritin (µg/l)			
At baseline	14.52±5.22	13.79±4.69	0.182
At 3 weeks	65.15±19.92	93.73±20.33	<0.0001
Change from baseline to 3 weeks	50.63±7.11	79.94±9.38	<0.0001
P-value^b^	<0.0001	<0.0001	
At 6 weeks	79.10±31.34	110.99±36.70	<0.0001
Change from baseline to 6 weeks	64.58±11.67	97.20±14.29	<0.0001
P-value^b^	<0.0001	<0.0001	

Data are expressed as the mean ± standard deviation or as number (%). Data were analyze using the

^a^unpaired t-test or a

^b^paired samples t-test. FCM, ferric carboxymaltose.

**Table III:** Changes in hemoglobin level among subgroups based on severity of anemia.

Variable	Iron sucrose (n=176)	FCM (n=158)	P-value^a^
Moderate anemia (n=104)			
Number of patients	57 (54.8)	47 (45.2)	
Hemoglobin level (g/dl)			
At baseline	8.24±1.40	7.98±1.45	0.355
At 3 weeks	10.47±1.62	10.86±1.27	0.181
Change from baseline to 3 weeks	2.23±0.37	2.88±0.52	<0.0001
P-value^b^	<0.0001	<0.0001	
At 6 weeks	10.94±0.69	11.84±1.49	<0.0001
Change from baseline to 6 weeks	2.70±0.16	3.90±0.76	<0.0001
P-value^b^	<0.0001	<0.0001	
Severe anemia (n=230)			
Number of patients	119 (51.7)	111 (48.3)	
Hemoglobin level (g/dl)			
At baseline	6.44±1.57	6.28±1.50	0.430
At 3 weeks	8.77±1.56	9.96±1.36	<0.0001
Change from baseline to 3 weeks	2.33±0.59	3.68±0.74	<0.0001
P-value^b^	<0.0001	<0.0001	
At 6 weeks	9.74±0.73	10.94±1.53	<0.0001
Change from baseline to 6 weeks	3.30±0.38	4.66±0.77	<0.0001
P-value^b^	<0.0001	<0.0001	

Data are expressed as the mean ± standard deviation or as number (%). Data were analyze using the

^a^unpaired t-test or a

^b^paired samples t-test. FCM, ferric carboxymaltose.

**Table IV:** Drug-related adverse events observed during and after the intravenous iron therapy.

Adverse event	Iron sucrose (n=176)	FCM (n=158)	P-value^a^
Local pain/burning/irritation at injection site	13 (7.4)	7 (4.4)	0.365
Swelling at injection site	12 (6.8)	6 (3.8)	0.328
Musculoskeletal/arthralgia	9 (5.1)	5 (3.2)	0.424
Headache/dizziness/giddiness	7(4)	3 (1.9)	0.344
Transient hypotension	7(4)	4 (2.5)	0.549
Nausea/vomiting	7(4)	3 (1.9)	0.344
Breathlessness/shivering	5 (2.8)	4 (2.5)	1.000
Severe anaphylactic reaction	0 (0)	0 (0)	1.000
Total no. of women reporting any adverse event	20 (11.4)	11(7)	0.232

Data are presented as number (%).

^a^Data were analyzed using the Chi-squared test or Fisher's exact test, as applicable. FCM, ferric carboxymaltose.

## Data Availability

The data generated in the present study may be requested from the corresponding author.
